# Inhibition of arabidopsis hypocotyl elongation by jasmonates is enhanced under red light in phytochrome B dependent manner

**DOI:** 10.1007/s10265-012-0509-3

**Published:** 2012-07-25

**Authors:** Jing Chen, Kohei Sonobe, Narihito Ogawa, Shinji Masuda, Akira Nagatani, Yuichi Kobayashi, Hiroyuki Ohta

**Affiliations:** 1 Department of Biological Sciences, Graduate School of Bioscience and Biotechnology, Tokyo Institute of Technology, 4259-B-65 Nagatsuta-cho, Midori-ku, Yokohama, 226-8501 Japan; 2 Department of Biomolecular Engineering, Graduate School of Bioscience and Biotechnology, Tokyo Institute of Technology, 4259-B-52 Nagatsuta-cho, Midori-ku, Yokohama, 226-8501 Japan; 3 Center for Biological Resources and Informatics, Graduate School of Bioscience and Biotechnology, Tokyo Institute of Technology, 4259-B-65 Nagatsuta-cho, Midori-ku, Yokohama, 226-8501 Japan; 4 Department of Botany, Graduate School of Science, Kyoto University, Sakyo-ku, Kyoto, 606-8502 Japan

**Keywords:** Jasmonate, Hypocotyl elongation, phyB

## Abstract

Jasmonates are phytohormones derived from oxygenated fatty acids that regulate a broad range of plant defense and developmental processes. In Arabidopsis, hypocotyl elongation under various light conditions was suppressed by exogenously supplied methyl jasmonate (MeJA). Moreover, this suppression by MeJA was particularly effective under red light condition. Mutant analyses suggested that SCF^COI1^-mediated proteolysis was involved in this function. However, MeJA action still remained in the *coi1* mutant, and (+)-7-iso-JA-L-Ile, a well-known active form of jasmonate, had a weaker effect than MeJA under the red light condition, suggesting that unknown signaling pathway are present in MeJA-mediated inhibition of hypocotyl elongation. EMS mutant screening identified two MeJA-insensitive hypocotyl elongation mutants, *jasmonate resistance long hypocotyl 1* (*jal1*) and *jal36*, which had mutations in the phytochrome B (*PHYB*) gene. These analyses suggested that inhibition of hypocotyl elongation by jasmonates is enhanced under red light in phyB dependent manner.

**Electronic supplementary material:**

The online version of this article (doi:10.1007/s10265-012-0509-3) contains supplementary material, which is available to authorized users.

## Introduction

Jasmonic acid and its derivates, collectively called jasmonates, are members of a plant hormone class derived from plastid membrane lipids. Jasmonates trigger senescence and inhibit root growth (Ueda and Kato 1980; Leshem 1988; Staswick et al. 1992). Wound-responsive proteins in tomato can be induced by methyl jasmonate (MeJA) treatment (Farmer and Ryan 1992). To date, jasmonates are considered important plant hormones with various roles in plant defense and diverse developmental processes (Turner 2007; Koo and Howe 2009).

In Arabidopsis, several enzymes including allene oxide synthase (AOS) and 12-oxo-phytodienoic acid reductase (OPR3) are involved in the biosynthesis of jasmonic acid (Schaller et al. 2000; Stintzi and Browse 2000; von Malek et al. 2002). *Jasmonate resistance 1* (*jar1*), which encodes a JA-amino synthetase, was isolated based on root growth inhibition by exogenously supplied jasmonates (Staswick et al. 1992, 2002; Staswick and Tiryaki 2004). The enzyme catalyzes the formation of jasmonoyl-l-isoleucine (JA-Ile) from jasmonic acid. Subsequently, (+)-7-iso-jasmonoyl-l-isoleucine ((+)-7-iso-JA-L-Ile) was found to be the bioactive jasmonate in Arabidopsis (Fonseca et al. 2009). Three other essential jasmonate-insensitive mutants (*coronatine insensitive 1* (*coi1*), *jasmonate insensitive 1* (*jin1*), and *jasmonate*-*insensitive 3* (*jai3*)) were also identified by the effect of jasmonate on root elongation (Feys et al. 1994; Berger et al. 1996). *COI1* encodes an E3 ubiquitin ligase that is part of the SCF (Skip/Cullin/Fbox) E3 ubiquitin ligase complexes (SCF^COI1^), which degrades target proteins and suppresses downstream gene transcription (Xie et al. 1998). *JIN1* encodes MYC2, a jasmonate responsive basic helix-loop-helix transcription factor that regulates transcription of various jasmonate-regulated genes (Lorenzo et al. 2004; Dombrecht et al. 2007; Pozo et al. 2008). After 10 years of COI1 research, 12 jasmonate ZIM domain proteins (JAZs; JAI3 and its 11 analogs) were identified as the targets of SCF^COI1^ (Chini et al. 2007; Thines et al. 2007; Yan et al. 2007). A central jasmonate signaling perception and transduction model was established based on the JA-Ile-mediated binding of JAZ and COI1 (Farmer 2007). This model starts with JA-Ile-dependent binding of SCF^COI1^ to JAZ proteins, which leads to SCF^COI1^-mediated degradation of JAZ repressors, releasing transcription factors like MYC2 and activates downstream gene expression (Chini et al. 2007; Thines et al. 2007; Fernández-Calvo et al. 2011). Furthermore, (+)-7-iso-JA-L-Ile synthesized by JAR1 is an exclusive signaling compound mediating JAZ and COI1 interaction but not other optical isomers (Fonseca et al. 2009).

JAs are also significant signals in photomorphogenesis. A *MYC2* mutation causes a shorter hypocotyl under continuous blue light conditions, suggesting that MYC2, which binds to JAZs, acts as a negative regulator of blue light-mediated photomorphogenesis (Yadav et al. 2005). Furthermore, MYC2 binds to the promoter region of *SUPPRESSOR OF PHYTOCHROME A1*, an important negative regulator of photomorphogenesis (Gangappa et al. 2010). The *fin 219* mutant exhibits a far red-specific long hypocotyl compared to the wild type and has an epigenetic mutation of *JAR1*, which is a link between phytochrome A (phyA) and the downstream regulator constitutive photomophogenic 1 (COP1) in light-mediated control of Arabidopsis development (Hsieh et al. 2000; Wang et al. 2011). In rice, OsJAR1 is involved in both jasmonate and phytochrome signaling (Riemann et al. 2008). COI1 is necessary for far-red light-induced transcription factor expression, and *coi1*-*16* flowers earlier than the wild type, suggesting that COI1 is involved in both the phyA- and phytochrome B (phyB)-dependent light signaling pathways (Robson et al. 2010).

Several photomorphogenesis-related genes are involved in jasmonate signaling. A phytochrome chromophore mutant *hy1* overproduces jasmonates and elevates expression of jasmonate-responsive genes (Zhai et al. 2007). A phyA mutation blocks jasmonate-mediated expression of *VEGETATIVE STORAGE PROTEIN 1* (*VSP1*), a typical jasmonate responsive gene, under dark or far-red light conditions (Robson et al. 2010). The *PHYTOCHROME AND FLOWERING TIME1* (*PFT1*) gene, which encodes MEDIATOR25, is a key regulator of the jasmonate-dependent defense response in Arabidopsis. Impairment of this gene causes decreased expression of *MYC2*, *OPR3, VSP*, and *PLANT DEFENSIN 1.2* (*PDF1.2*) after jasmonate treatment (Kidd et al. 2009).

To investigate the crosstalk between jasmonate and light signaling, jasmonate function in Arabidopsis hypocotyl elongation under various light conditions was examined. Hypocotyl elongation was suppressed by MeJA particularly under red light condition. Several jasmonate-resistant mutants were identified in screens of MeJA effects on hypocotyl development. Two of these mutants had a phyB mutation, demonstrating its importance in jasmonate signaling.

## Materials and methods

### Plant materials and growth conditions

Wild-type (Col-0, Ler, Ws) and mutant plants used in this study were obtained from *Arabidopsis Biological Resource Centre*, except the *coi1* (see below). Seeds were surface-sterilized and sown on Murashige–Skoog medium agar plates with 1 % (w/v) sucrose (except in Figure S3). Seeds were incubated for 2 days at 4 °C, and germination was initiated by 4 h of irradiation with white light before transferring to the different growth conditions. Seedlings were grown at 23 °C with continuous different light conditions (provided by LEDs). For dark treatment, plates were wrapped in two layers of aluminum foil. Plates were placed in a vertical orientation. For JA treatment, seeds were sown on the plates containing different concentrations jasmonates. (−)-JA-L-Ile and (+)-7-iso-JA-L-Ile were chemically synthesized as described (Ogawa and Kobayashi 2012).

### Root and hypocotyl length measurement

Root and hypocotyl length was measured according to Kobayashi et al. (2009) with minor modification. Briefly, seedling growth was captured on a digital camera (Nikon D80), and primary root length and hypocotyl length were measured with image analyzing software (ImageJ; http://rsb.info.nih.gov/ij).

### Isolation of the *coi1*-*16* single mutant

The temperature-dependent COI1 single mutant *coi1*-*16s* was screened from a F3 seed pool of crosses between Col and *coi1*-*16* (kindly provided by John G. Turner). MeJA-insensitive plant genomes were analyzed with polymerase chain reaction (PCR) and *Aci*I digestion (Westphal et al. 2008). F3 seeds were screened by phenotype and sequencing of *COI1* and *PENETRATION2* (*PEN2*) loci to confirm a single point mutation in *COI1* locus. Both *coi1*-*16* and *coi1*-*16* single mutant (*coi1*-*16s*) were maintained at 16 °C to obtain seeds.

### Ethyl methanesulfonate treatment and screening

Ethyl methanesulfonate (EMS)-mutagenized M2 seeds of the Col-0 wild-type accession were obtained as described by Leyser et al. publication (from TAIR). Approximately 20,000 M2 seeds were germinated and grown vertically on medium containing 50 μM MeJA for 10 days. Seedlings with longer hypocotyls were selected as *jal* mutant candidates and directly transplanted into soil. M3 seeds from the putative mutants were confirmed as having a long hypocotyl phenotype on medium containing 10 and 50 μM MeJA. The original *jal* mutant was backcrossed to Col-0 for two generations, and the resulting homozygous progenies were used in this study.

### Protein extraction and western blotting analysis

PHYB extraction and western blotting were performed as described by Leivar et al. (2008). Generally, for PHYB immunoblots, total protein was extracted using extraction buffer [100 mM MOPS (pH 7.6), 2 % (w/v) SDS, 50 mM metabisulfite, 10 % (v/v) glycerol, 4 mM EDTA, 2 μg/L aprotinin, 3 μg/L leupeptin, 1 μg/L pepstatin, and 2 mM phenylmethanesulfonylfluoride]. Total protein was quantified using a Protein DC kit (Bio-Rad), and 40 μM β-mercaptoethanol was added just before loading. Monoclonal antibodies anti-BA02 specific to the C-terminal domain of PHYB was used to immunodetect PHYB (Matsushita et al. 2003). Anti-mouse horseradish peroxidase (Thermo scientific; http://www.perbio.com/) was used as a secondary antibody, and ECL-plus western blotting detection system (GE healthcare Amersham; http://www.gelifesciences.com/) were used for detection.

## Results

### Jasmonates suppress hypocotyl elongation in a SCF^COI1^-dependent pathway

**Fig. 1:**
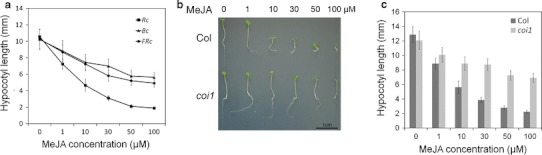
Jasmonates suppress hypocotyl elongation under various light conditions. **a** Hypocotyl lengths of 6-day-old Col seedlings with 0, 1, 10, 30, 50, or 100 μM MeJA treatment on MS medium (with 1 % sucrose) under 50–60 μmol m^−2^ s^−1^ Rc, 2–5 μmol m^−2^ s^−1^ Bc or 0.02–0.05 μmol m^−2^ s^−1^ FRc. **b** Photograph of 6-day-old Col and *coi1*-*16s* seedlings grown on MS medium (with 1 % sucrose) with different concentrations of MeJA under 30–50 μmol m^−2^ s^−1^ Rc. **c** Hypocotyl lengths of 6-day-old Col and *coi1*-*16s* mutant seedlings with 0, 1, 10, 30, 50, or 100 μM MeJA treatment on MS medium (with 1 % sucrose) under 30–50 μmol m^−2^ s^−1^ Rc. For **a** and **c** data are the mean ± SD (*n* = 20 seedlings per genotype)

Hypocotyl elongation is controlled differently by various light conditions such as dark, red, blue, and far-red light. Jasmonates are inhibitors of regeneration and suppress root development in a SCF^COI1^-dependent manner (Xie et al. 1998). Here, the effects of jasmonates on Arabidopsis hypocotyl development under continuous red light (Rc), blue light (Bc), far red light (FRc) and dark were analyzed. To compare the jasmonate effect among different light conditions, we firstly established appropriate light intensity to get a similar hypocotyl length in different light conditions without jasmonate. Otherwise, hypocotyl inhibition by Bc and FRc are much stronger than Rc, and resulting shorter hypocotyl may prevent to correctly compare jasmonate action under various light conditions. Exogenous MeJA inhibited hypocotyl elongation in the wild type in a concentration dependent manner in all four light conditions (Fig. 1a, S1). However, under Rc, MeJA showed much stronger effect than those under Bc, FRc (Fig. 1a) and dark (Figure S1A) (*P* < 0.01 in all concentrations). These results suggest that some jasmonate signaling pathway was activated under Rc.

Jasmonate signaling is largely dependent of COI1, a central component of jasmonate signaling. *coi1*-*1* a null mutant of COI1 is generally utilized to analyze COI1-dependence in various jasmonate-mediated physiological events. However, *coi1*-*1* is male-sterile, and thus, utilization of this mutant for detailed analysis of hypocotyl suppression by jasmonates under various light conditions is quite difficult. In order to analyze the COI1 function on hypocotyl suppression by jasmonate, a temperature-dependent *COI1* single mutant (*coi1*-*16s*) was isolated from F3 seeds of *coi1*-*16* (Ellis and Turner 2002; Westphal et al. 2008) back-crossed with wild type (Col) to eliminate another mutation at *PENETRATION2* (*PEN2*) in *coi1*-*16*, since this additional mutation may cause unknown effects on the hypocotyl elongation. Genome DNA sequences at the mutation sites of *COI1* and *PEN2* were determined and confirmed that *coi1*-*16s* have a mutation at *COI1* but not at *PEN2*. Temperature-dependency of *coi1*-*16s* was examined in Figure S2 and it showed similar results with *coi1*-*16* which is insensitive to MeJA treatment under 23 °C but not 16 °C. In contrast to the wild type, the effect of MeJA was partially abolished in the *coi1*-*16s* hypocotyl because of impaired COI1-dependent jasmonate signaling under Rc (Fig. 1b, c) and dark (Figure S1A). These results indicate that jasmonates suppress hypocotyl elongation under dark and Rc in a SCF^COI1^-dependent manner. However, under Rc, almost half of the inhibition still remained even in the *coi1*-*16s* mutant (Fig. 1c), suggesting that a COI1 independent signaling is also present in MeJA-mediated inhibition of hypocotyl elongation under red light.

**Fig. 2:**
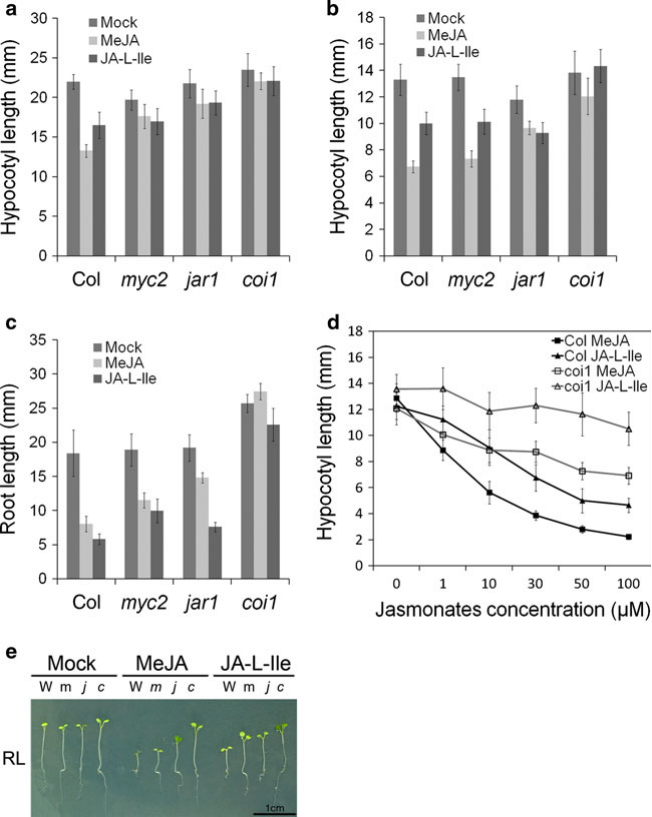
Effect of jasmonoyl isoleucine on hypocotyl elongation. **a**, **b** Hypocotyl length of 6-day-old seedlings of Col, *myc2*, *jar1*, and *coi1*-*16s* grown on MS medium (with 1 % sucrose) containing 10 μM MeJA or 10 μM (+)-7-iso-JA-L-Ile under dark (**a**) or 30–50 μmol m^−2^ s^−1^ Rc (**b**). **c** Root length of 6-day-old seedlings of Col, *myc2*, *jar1*, and *coi1*-*16s* grown on MS medium (with 1 % sucrose) containing 10 μM MeJA or 10 μM (+)-7-iso-JA-L-Ile under continuous white light. **d** Hypocotyl lengths of 6-day-old Col and *coi1*-*16s* mutant seedlings with 0, 1, 10, 30, 50, or 100 μM MeJA (*square*) and (+)-7-iso-JA-L-Ile (*triangle*) treatment on MS medium (with 1 % sucrose) under 30–50 μmol m^−2^ s^−1^ Rc. JA-L-Ile indicates (+)-7-iso-JA-L-Ile. **e** Photograph of 6-day-old seedlings of Col (*W*), *myc2* (*m*), *jar1* (*j*), and *coi1*-*16s* (*c*) grown on MS medium (with 1 % sucrose) containing 10 μM MeJA or 10 μM (+)-7-iso-JA-L-Ile under 30–50 μmol m^−2^ s^−1^ Rc. For **a**–**d** data are the mean ± SD (*n* = 20 seedlings per genotype)

To determine whether the MYC2, downstream transcription factor of jasmonate signaling, was involved in hypocotyl suppression, hypocotyl elongation was analyzed in the *myc2* mutant. In the dark, *MYC2* knockout resulted in resistance to MeJA-induced hypocotyl inhibition, similar to root elongation (Fig. 2a, c). However, under Rc with MeJA addition, *myc2* hypocotyl elongation was suppressed to a similar extent as the wild type (Fig. 2b, e). These results suggest that MYC2 is required for JA signaling during hypocotyl inhibition under dark but not Rc conditions.

We considered whether the active jasmonate conjugate on hypocotyl inhibition under red light was different from root suppression. Jasmonoyl-l-isoleucine, which is biosynthesized from jasmonic acid by JAR1 (Staswick et al. 1992, 2002; Staswick and Tiryaki 2004) was analyzed by the effect on hypocotyl elongation. The isomer (+)-7-iso-JA-L-Ile was identified as an active form that triggers binding of COI1 and JAZ proteins (Fonseca et al. 2009). In this study, the *jar1* mutant was insensitive to MeJA, with the hypocotyl elongating longer than the wild type in both the dark and red light after MeJA addition (Fig. 2a, b, e). Furthermore, (+)-7-iso-JA-L-Ile suppressed root development in the wild type and *jar1* which was largely insensitive to MeJA (Fig. 2c), validating previous results (Fonseca et al. 2009) and supporting the idea that (+)-7-iso-JA-L-Ile is a ligand of COI1 in root elongation. Unexpectedly, (+)-7-iso-JA-L-Ile was less effective on wild-type hypocotyl development than MeJA under Rc (Fig. 2b, d) and dark (Fig. 2a). It was shown that (+)-7-iso-JA-L-Ile suppressed hypocotyl elongation under Rc in a concentration dependent manner; but it showed weaker effect than MeJA on Col in all concentrations (*P* < 0.01 in all concentrations) even at 100 μM (Fig. 2d). And in *coi1*-*16s* mutant, (+)-7-iso-JA-L-Ile function was almost abolished under Rc (Fig. 2d). From these results, we suggest that MeJA itself or an unknown jasmonate-related compound derived from MeJA may be responsible for regulating downstream signaling and affects hypocotyl elongation under red light.

### Identification of mutants with jasmonate-insensitive hypocotyl elongation

**Fig. 3:**
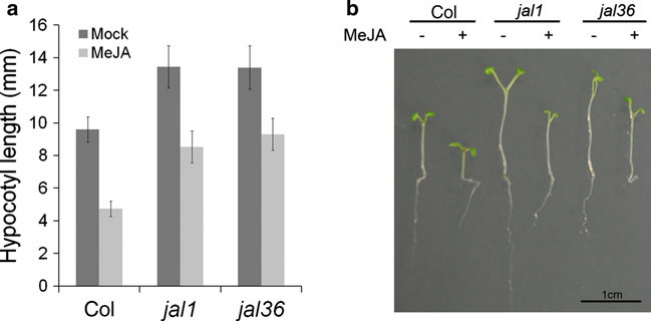
Phenotype of screened *jal1* and *jal36.* Hypocotyl length (**a**) and photograph (**b**) of 6-day-old Col, *jal1*, and *jal36* seedlings grown on MS medium (with 1 % sucrose) with (+) or without (−) 10 μM MeJA under 30–50 μmol m^−2^ s^−1^ Rc. Data are the mean ± SD (*n* = 15 seedlings per genotype)

To uncover the COI1-independent jasmonate signaling pathway involved in hypocotyl elongation, mutants were screened for jasmonate suppression of hypocotyl elongation. EMS mutant seeds (M2) were screened on MeJA-containing MS medium under constant red light. From 20,000 M2 seeds, 56 mutants displayed longer hypocotyls but similar root lengths compared to the wild type under Rc. Among them, *jasmonate long hypocotyl 1* (*jal1*) and *jal36* were confirmed as partially insensitive to MeJA for hypocotyls elongation (but not for root elongation) under red light (Fig. 3). Although *jal1* and *jal36* exhibited longer hypocotyls than the wild type even in the absence of jasmonates, MeJA was relatively less effective on the mutants than on wild-type plants (Fig. 3, S1F). These results indicate that the mutated gene(s) of these two mutants play a pivotal role in jasmonate signaling during hypocotyl elongation.

**Fig. 4:**
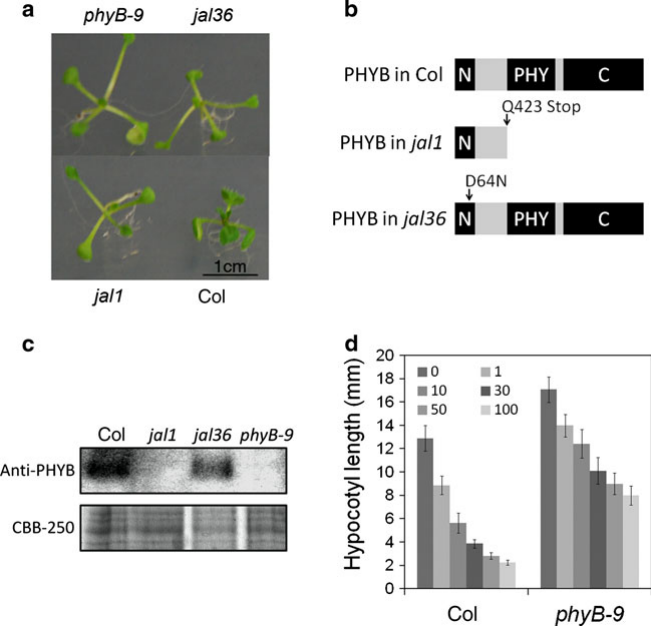
Identification of mutant loci in *jal1* and *jal36*. **a** Photograph of 10-day-old Col, *jal1*, *jal36*, and *phyB*-*9* seedlings grown on MS medium under white light. **b** Illustration of the *PHYB* locus of *jal1* and *jal36*. Schematic of phyB is shown by N-terminal extension region (N) (residues 1–102), the phytochrome domain (PHY) (residues 444–623) and C-terminal region (C) by *black box*; the *arrows* indicate the point mutation site in *jal1* and *jal36*. **c** Western blots of 10-day-old Col, *jal1*, *jal36*, and *phyB*-*9* using anti-PHYB as a primary antibody. Total protein was stained by CBB-250. **d** Hypocotyl length of 6-day-old Col and *phyB*-*9* seedlings grown on MS medium (with 1 % sucrose) with or without 10 μM MeJA under 30–50 μmol m^−2^ s^−1^ Rc

With white light, *jal1* and *jal36* had longer hypocotyls and petioles, and smaller blades than the wild type (Figs. 3, 4a). These phenotypes were consistent with the known *phyB*-*9* mutant, which lacks a red light receptor phyB (Somers et al. 1991). We hypothesized that *jal1* and *jal36* mutants had a loss of function of phyB, and hence, their *PHYB* loci were analyzed. Sequencing revealed that *PHYB* loci in both *jal1* and *jal36* possessed a point mutation resulting in a truncated protein and a D64 N mutated protein, respectively (Fig. 4b). Further analysis with western blotting was performed to clarify the PHYB content. Like *phyB*-*9*, the stop codon generated by a point mutation caused elimination of phyB in *jal1*, whereas PHYB was detected in *jal36* (Fig. 4c). However, according to Oka et al. (2008), the D64N mutation probably caused deactivation of the phyB protein in Arabidopsis. To confirm that the impairment of jasmonate signaling under Rc was actually due to phyB knockout, MeJA sensitivity of the *phyB*-*9* mutant under Rc was tested. As expected, *phyB*-*9* hypocotyls after MeJA addition were relatively longer than in the wild type (Fig. 4d, S1B), which is consistent with observations in *jal1* and *jal36* (Fig. 3a, S1F). It indicates that the phyB mutation in *jal1*, *jal36*, and *phyB*-*9* partially increased MeJA resistance in hypocotyl inhibition under red light. In contrast to *phyB* phenotype under red light, *phyA* mutant (Ler background *phyA*-*201*) did not show MeJA insensitivity even under far-red light conditions (Figure S1C, S1D). This result rather showed that phyA mutation caused hypersensitive phenotype to jasmonate which is different with phyB, indicating complicated crosstalk between jasmonate and light signaling.

## Discussion

The plant defense-related phytohormone jasmonate inhibits Arabidopsis root development (Xie et al. 1998). Several important jasmonate signaling genes such as *COI1*, *JAR1*, *MYC2*, and *JAZ* were isolated by screening for the jasmonate effect on root development (Staswick et al. 1992; Feys et al. 1994; Berger et al. 1996). Here, we described another jasmonate effect on hypocotyl elongation under various light conditions.

### Unknown jasmonate signaling is involved in suppression of hypocotyl elongation under Rc

The hypocotyl elongation of endogenous jasmonate biosynthesis mutant *aos* was shown longer than wild type under low Rc or dark (*P* < 0.01) without MeJA, especially without sucrose. This indicates jasmonate signaling is important to the hypocotyl elongation (Figure S3). And the hypocotyl elongation (Fig. 1) in *coi1*-*16s* were suppressed by a high jasmonate concentration, which supports previous results showing the jasmonate-responsive gene expression of the *coi1*-*1* null mutant upon jasmonate treatment or wounding (Chung et al. 2008). Although basal or jasmonate-mediated expression of these genes in *coi1* is much lower than in the wild type, these genes still weakly respond to jasmonates even in the *coi1* null mutant (Chung et al. 2008). Although *coi1*-*16* is not a null mutant, but light signaling-related phenotype of the *coi1*-*16* mutant has been analyzed and similar results to *coi1*-*1* null mutant were shown (Robson et al. 2010). We suggest that a COI1-independent jasmonate signaling is also present in hypocotyl elongation. (+)-7-iso-JA-L-Ile, which is synthesized by JAR1 from jasmonic acid (Staswick and Tiryaki 2004) is the bioactive form that inhibits root development (Fonseca et al. 2009). In contrast to its isomer (−)-JA-L-Ile, (+)-7-iso-JA-L-Ile is the true ligand of the jasmonate receptor SCF^COI1^-JAZ protein complex (Fonseca et al. 2009), which mediates binding between COI1 and JAZ proteins and regulates downstream gene expression. Indeed, (+)-7-iso-JA-L-Ile had the strongest effect on root development in both the wild type and *jar1* (Fig. 2c). MeJA, however, showed a stronger effect on hypocotyl elongation (Fig. 2a, b). (+)-7-iso-JA-L-Ile treatment with a higher concentration also showed weaker effect compared to the MeJA under Rc (Fig. 2d) and dark. Moreover, jasmonic acid, a less volatile compound than MeJA also showed stronger effect than (+)-7-iso-JA-L-Ile (Figure S1E). These results suggest that different uptake of MeJA and (+)-7-iso-JA-L-Ile between root and shoot or volatility of the MeJA is not the reason for the different effects of the jasmonates on shoot and root. There is an unknown jasmonate signaling involved in the hypocotyl elongation suppression. Furthermore, the mutation on *COI1* almost totally abolished the (+)-7-iso-JA-L-Ile function under red light (Fig. 2d). Although we cannot completely exclude a possibility that *coi1*-*16s* is leaky even under normal growth conditions, we speculate that a COI1-independent pathway is present and mediated by an unknown jasmonate other than (+)-7-iso-JA-L-Ile which has a central role in COI1 dependent jasmonate signaling. It should be noted that (+)-7-iso-JA-L-Ile showed weaker effect than MeJA even under dark (Fig. 2a). This result together with Figure S1A and S1B indicates that COI1-independent and MeJA-specific response is present even under dark. In this aspect, it is also of interest that under Rc, hypocotyl elongation of *myc2* mutant is more sensitive to MeJA treatment than under dark (Figure S2A, S2B). MYC2 is known to be a major jasmonate signaling component, and mutation on MYC2 leads to insensitivity to MeJA treatment for root elongation. However, the present results indicate that MYC2 is more important for jasmonate function under dark than Rc. Although redundant effect of MYC2, MYC3 and MYC4 might be responsible for the sensitivity to MeJA under red light (Fernández-Calvo et al. 2011), our results suggest that under Rc, jasmonate inhibit hypocotyl elongation in both COI1 dependent and independent ways, and MYC2 may participate in the COI1 dependent signaling under all light conditions. COI1 independent pathway may be enhanced under red light, which is probably mediated by MeJA or other unknown jasmonate derived from MeJA.

### Phytochrome B is involved in jasmonate signaling under Rc

Furthermore, in Rc, MeJA showed stronger effect on hypocotyl elongation (Fig. 1a) than other light conditions. It suggests that except the general signaling pathway in all light conditions, another unknown signaling pathway was activated under Rc. The *phyB*-*9* was shown to be partially insensitive to jasmonate treatment under red light compared to Col. As a red light receptor, phyB play a pivotal role in the red light signaling. These indicate that phyB is involved in jasmonate signaling leading to the stronger effect of jasmonate on hypocotyl elongation under red light. As shown under Rc, jasmonate inhibits hypocotyl elongation in both COI1 dependent and independent ways. Moreover, it was shown that hypocotyl lengths in MeJA-treated *phyB*-*9* mutants under Rc were quite similar to those in wild type under dark (Figure S1A, S1B). We suggest that under Rc, well known (COI1-dependent) jasmonate signaling and the phyB-mediated signaling were combined and suppressed hypocotyl elongation in an additive manner. We speculate that phyB enhances COI1-independent jasmonate signaling under red light.

## Conclusion

In summary, the hypocotyl elongation of Arabidopsis was suppressed by exogenously supplied MeJA; SCF^COI1^-mediated proteolysis and phyB are involved in this function. (+)-7-iso-JA-L-Ile showed a weaker effect than MeJA, suggesting that suppression of hypocotyl elongation was controlled by a different pathway from roots. Two mutants isolated by screening for the MeJA effect on hypocotyl elongation under Rc had mutations in *PHYB* locus. Further analysis indicated that phyB is involved in jasmonate effect on suppression of hypocotyl elongation under Rc. Jasmonate signaling interacts with red light signaling through unknown signaling pathway possibly in (+)-7-iso-JA-L-Ile independent manner. This (+)-7-iso-JA-L-Ile- and COI1-independent signaling under red light might be mediated by phyB.

## Electronic supplementary material

Below is the link to the electronic supplementary material.

Supplementary material 1 (PDF 641 kb)

## References

[CR1] Berger S, Bell E, Mullet JE (1996). Two methyl jasmonate-insensitive mutants show altered expression of AtVsp in response to methyl jasmonate and wounding. Plant Physiol.

[CR3] Chini A, Fonseca S, Fernandez G, Adie B, Chico JM, Lorenzo O, Garcia-Casado G, Lopez-Vidriero I, Lozano FM, Ponce MR, Micol JL, Solano R (2007). The JAZ family of repressors is the missing link in jasmonate signaling. Nature.

[CR5] Chung HS, Koo AJ, Gao X, Jayanty S, Thines B, Jones AD, Howe GA (2008). Regulation and function of Arabidopsis JASMONATE ZIM-domain genes in response to wounding and herbivory. Plant Physiol.

[CR6] Dombrecht B, Xue GP, Sprague SJ, Kirkegaard JA, Ross JJ, Reid JB, Fitt GP, Sewelam N, Schenk PM, Manners JM, Kazan K (2007). MYC2 differentially modulates diverse jasmonate-dependent functions in Arabidopsis. Plant Cell.

[CR7] Ellis C, Turner JG (2002). A conditionally fertile coil allele indicates cross-talk between plant hormone signalling pathways in Arabidopsis thaliana seeds and young seedlings. Planta.

[CR8] Farmer EE (2007). Plant biology: jasmonate perception machines. Nature.

[CR9] Farmer EE, Ryan CA (1992). Octadecanoid precursors of jasmonic acid activate the synthesis of wound-inducible proteinase inhibitors. Plant Cell.

[CR26] Fernández-Calvo P, Chini A, Fernández-Barbero G, Chico JM, Gimenez-Ibanez S, Geerinck J, Eeckhout D, Schweizer F, Godoy M, Franco-Zorrilla JM, Pauwels L, Witters E, Puga MI, Paz-Ares J, Goossens A, Reymond P, De Jaeger G, Solano R (2011) The Arabidopsis bHLH transcription factors MYC3 and MYC4 are targets of JAZ repressors and act additively with MYC2 in the activation of jasmonate responses. Plant Cell 23:701–715 10.1105/tpc.110.080788 PMC3077776 21335373

[CR10] Feys B, Benedetti CE, Penfold CN, Turner JG (1994). Arabidopsis mutants selected for resistance to the phytotoxin coronatine are male sterile, insensitive to methyl jasmonate, and resistant to a bacterial pathogen. Plant Cell.

[CR11] Fonseca S, Chini A, Hamberg M, Adie B, Porzel A, Kramell R, Miersch O, Wasternack C, Solano R (2009). (+)-7-iso-Jasmonoyl-l-isoleucine is the endogenous bioactive jasmonate. Nat Chem Biol.

[CR12] Gangappa SN, Prasad VB, Chattopadhyay S (2010). Functional interconnection of MYC2 and SPA1 in the photomorphogenic seedling development of Arabidopsis. Plant Physiol.

[CR100] Hsieh HL, Okamoto H, Wang M, Ang LH, Matsui M, Goodman H, Deng XW (2000) FIN219, an auxin-regulated gene, defines a link between phytochrome A and the downstream regulator COP1 in light control of Arabidopsis development. Gene Dev 14:1958–1970 PMC316819 10921909

[CR14] Kidd BN, Edgar CI, Kumar KK, Aitken EA, Schenk PM, Manners JM, Kazan K (2009). The mediator complex subunit PFT1 is a key regulator of jasmonate-dependent defense in Arabidopsis. Plant Cell.

[CR15] Kobayashi K, Awai K, Nakamura M, Nagatani A, Masuda T, Ohta H (2009). Type-B monogalactosyldiacylglycerol synthases are involved in phosphate starvation-induced lipid remodeling, and are crucial for low-phosphate adaptation. Plant J.

[CR16] Koo AJ, Howe GA (2009). The wound hormone jasmonate. Phytochemistry.

[CR17] Leivar P, Monte E, Al-Sady B, Carle C, Storer A, Alonso JM, Ecker JR, Quail PH (2008). The Arabidopsis phytochrome-interacting factor PIF7, together with PIF3 and PIF4, regulates responses to prolonged red light by modulating phyB levels. Plant Cell.

[CR18] Leshem YY (1988). Plant senescence processes and free radicals. Free Radical Bio Med.

[CR20] Lorenzo O, Chico JM, Sanchez-Serrano JJ, Solano R (2004). JASMONATE-INSENSITIVE1 encodes a MYC transcription factor essential to discriminate between different jasmonate-regulated defense responses in Arabidopsis. Plant Cell.

[CR22] Matsushita T, Mochizuki N, Nagatani A (2003). Dimers of the N-terminal domain of phytochrome B are functional in the nucleus. Nature.

[CR24] Ogawa N, Kobayashi Y (2012) Synthesis of the amino acid conjugates of epi-jasmonic acid. Amino Acids 42:1955–1966 10.1007/s00726-011-0925-z 21562820

[CR25] Oka Y, Matsushita T, Mochizuki N, Quail PH, Nagatani A (2008). Mutant screen distinguishes between residues necessary for light-signal perception and signal transfer by phytochrome B. PLoS Genet.

[CR27] Pozo MJ, Van Der Ent S, Van Loon LC, Pieterse CM (2008). Transcription factor MYC2 is involved in priming for enhanced defense during rhizobacteria-induced systemic resistance in *Arabidopsis thaliana*. New Phytol.

[CR28] Riemann M, Riemann M, Takano M (2008). Rice JASMONATE RESISTANT 1 is involved in phytochrome and jasmonate signalling. Plant Cell Environ.

[CR29] Robson F, Okamoto H, Patrick E, Harris SR, Wasternack C, Brearley C, Turner JG (2010). Jasmonate and phytochrome A signaling in Arabidopsis wound and shade responses are integrated through JAZ1 stability. Plant Cell.

[CR30] Schaller F, Biesgen C, Mussig C, Altmann T, Weiler EW (2000). 12-Oxophytodienoate reductase 3 (OPR3) is the isoenzyme involved in jasmonate biosynthesis. Planta.

[CR31] Somers DE, Sharrock RA, Tepperman JM, Quail PH (1991). The hy3 long hypocotyl mutant of arabidopsis is deficient in phytochrome B. Plant Cell.

[CR32] Staswick PE, Tiryaki I (2004). The oxylipin signal jasmonic acid is activated by an enzyme that conjugates it to isoleucine in Arabidopsis. Plant Cell.

[CR33] Staswick PE, Su W, Howell SH (1992). Methyl jasmonate inhibition of root growth and induction of a leaf protein are decreased in an *Arabidopsis thaliana* mutant. Proc Natl Acad Sci USA.

[CR34] Staswick PE, Tiryaki I, Rowe ML (2002). Jasmonate response locus JAR1 and several related Arabidopsis genes encode enzymes of the firefly luciferase superfamily that show activity on jasmonic, salicylic, and indole-3-acetic acids in an assay for adenylation. Plant Cell.

[CR120] Stintzi A, Browse J (2000) The Arabidopsis male-sterile mutant, opr3, lacks the 12-oxophytodienoic acid reductase required for jasmonate synthesis. Proc Natl Acad Sci USA 97:10625–10630 10.1073/pnas.190264497 PMC27075 10973494

[CR35] Thines B, Katsir L, Melotto M, Niu Y, Mandaokar A, Liu G, Nomura K, He SY, Howe GA, Browse J (2007). JAZ repressor proteins are targets of the SCF(COI1) complex during jasmonate signalling. Nature.

[CR36] Turner JG (2007). Stress responses: JAZ players deliver fusion and rhythm. Curr Biol.

[CR37] Ueda J, Kato J (1980). Isolation and identification of a senescence-promoting substance from wormwood (*Artemisia absinthium* L.). Plant Physiol.

[CR38] von Malek B, van der Graaff E, Schneitz K, Keller B (2002). The Arabidopsis male-sterile mutant dde2-2 is defective in the ALLENE OXIDE SYNTHASE gene encoding one of the key enzymes of the jasmonic acid biosynthesis pathway. Planta.

[CR39] Wang JG, Chen CH, Chien CT, Hsieh HL (2011). FAR-RED INSENSITIVE219 modulates CONSTITUTIVE PHOTOMORPHOGENIC1 activity via physical interaction to regulate hypocotyl elongation in Arabidopsis. Plant Physiol.

[CR40] Westphal L, Scheel D, Rosahl S (2008). The coi1-16 mutant harbors a second site mutation rendering PEN2 nonfunctional. Plant Cell.

[CR41] Xie DX, Feys BF, James S, Nieto-Rostro M, Turner JG (1998). COI1: an Arabidopsis gene required for jasmonate-regulated defense and fertility. Science.

[CR42] Yadav V, Mallappa C, Gangappa SN, Bhatia S, Chattopadhyay S (2005). A basic helix-loop-helix transcription factor in Arabidopsis, MYC2, acts as a repressor of blue light-mediated photomorphogenic growth. Plant Cell.

[CR43] Yan Y, Stolz S, Chetelat A, Reymond P, Pagni M, Dubugnon L, Farmer EE (2007). A downstream mediator in the growth repression limb of the jasmonate pathway. Plant Cell.

[CR44] Zhai Q, Li CB, Zheng W, Wu X, Zhao J, Zhou G, Jiang H, Sun J, Lou Y, Li C (2007). Phytochrome chromophore deficiency leads to overproduction of jasmonic acid and elevated expression of jasmonate-responsive genes in Arabidopsis. Plant Cell Physiol.

