# Supplementary material for: Inhibition of arabidopsis hypocotyl elongation by jasmonates is enhanced under red light in phytochrome B dependent manner
Source: J Plant Res. 2012 Jul 25;126(1):161–8. doi: 10.1007/s10265-012-0509-3 (PMC3530149; doi:10.1007/s10265-012-0509-3)
Supplement: Supplementary file 1 — Supplementary material 1 (PDF 641 kb) [file 10265_2012_509_MOESM1_ESM.pdf]

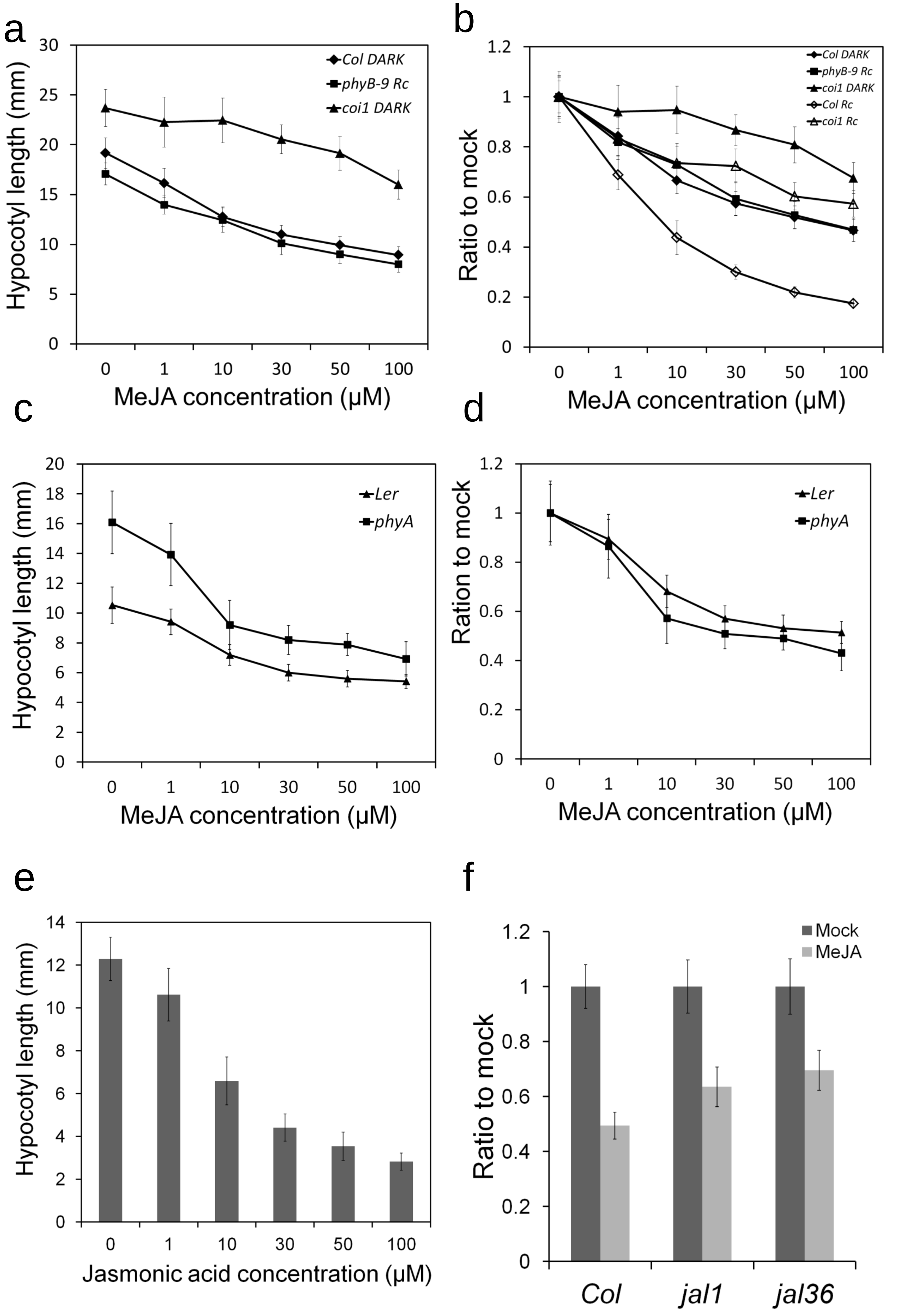
a
Hypocotyl length (mm)
30
25
20
15
10
5
0
MeJA concentration (μM)
0
1
10
30
50
100
Col DARK
phyB-9 Rc
coi1 DARK
b
Ratio to mock
1.2
1
0.8
0.6
0.4
0.2
0
MeJA concentration (μM)
Col DARK
phyB-9 Rc
coi1 DARK
Col Rc
coi1 Rc
c
Hypocotyl length (mm)
20
18
16
14
12
10
8
6
4
2
0
MeJA concentration (μM)
Ler
phyA
d
Ration to mock
MeJA concentration (μM)
Ler
phyA
e
Hypocotyl length (mm)
14
12
10
8
6
4
2
0
Jasmonic acid concentration (μM)
f
Ratio to mock
Mock
MeJA
Col
jal1
jal36

Figure S1. Jasmonates suppress hypocotyl elongation various light conditions.

a, Hypocotyl lengths of 6-d-old Col or *phyB-9, coi1* mutant seedlings with 0, 1, 10, 30, 50, or 100 μM MeJA treatment on MS medium under dark (Col, *coi1-16s*) or Rc (*phyB-9*).

b, Hypocotyl lengths ratio of 6-d-old Col or *phyB-9,coi1* mutant seedlings with 0, 1, 10, 30, 50, or 100 μM MeJA treatment on MS medium under dark (Col,*coi1-16s*) or Rc (*phyB-9, Col, coi1-16s*) compared to mock treatment.

c, Hypocotyl lengths of 6-d-old Ler or *phyA* mutant (*phyA-201*) seedlings with 0, 1, 10, 30, 50, or 100 μM MeJA treatment on MS medium under 0.02-0.05 $\mu molm^{-2}s^{-1}$ FRc.

d, Relative data of Figure S1c compared to Ler.

e, Hypocotyl lengths of 6-d-old Col seedlings with 0, 1, 10, 30, 50, or 100 μM jasmonic acid treatment on MS medium under 30-50 $\mu molm^{-2}s^{-1}$ Rc.

f, Relative data of Figure 3 compared to mock.

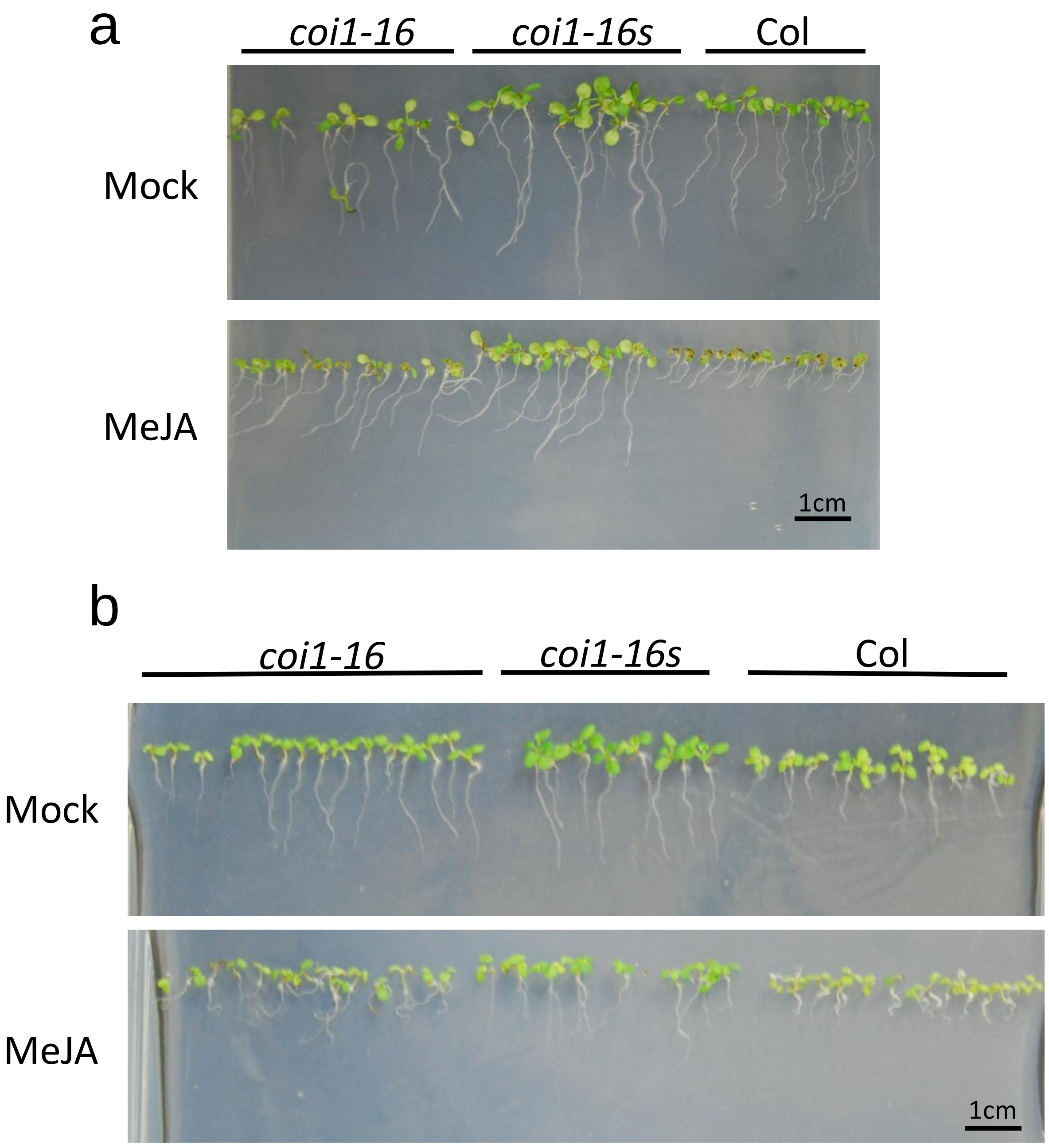

Figure S2. Photograph of Col , *coi1-16* and *coi1-16s* seedlings grown on MS medium with or without 50 μM MeJA under white light, at 23 degrees centigrade for 6 days (a), and at 16 degrees centigrade for 21 days (b)

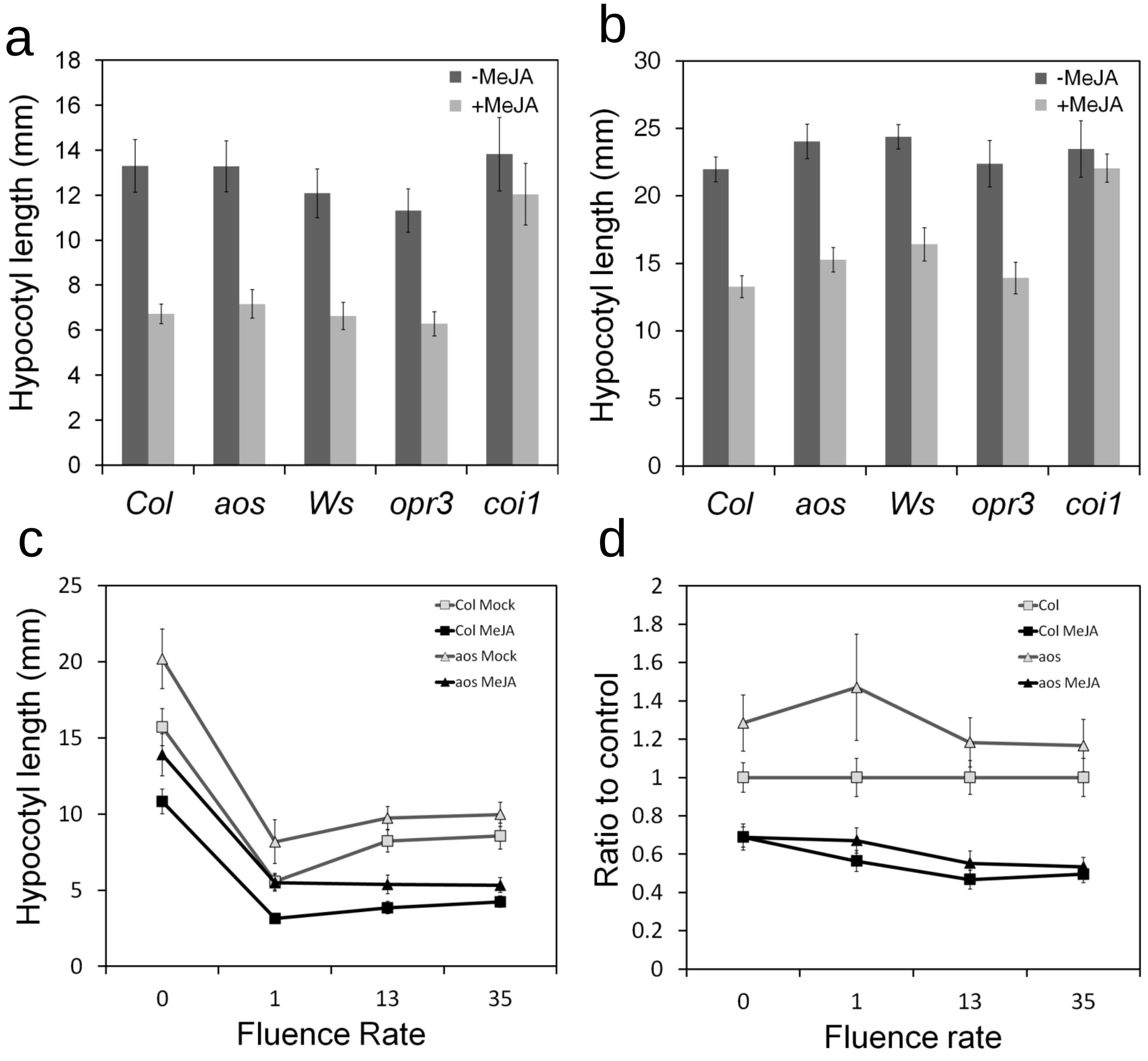

Figure S3

a, b, Hypocotyl length of 6-d-old seedlings of Col, *aos*, Ws, *opr3*, and *coi1-16s* grown on MS medium (with 1% sucrose) under 30-50 μmolm^{-2}s^{-1} Rc or dark conditions with or without 10 μM MeJA.

c, Hypocotyl lengths of 6-d-old Col and *aos* seedlings grown under 0, 1, 13, or 35 μmolm^{-2}s^{-1} Rc on MS medium (no sucrose) with or without 10 μM MeJA.

For a to c, data are the means ± S.D. (n = 15 seedlings per genotype).

d, Relative data of figure S3c compared to the Col mock for *aos* mock and Col MeJA, to *aos* mock for *aos* MeJA
